# Effect of radio-frequency electromagnetic radiations (RF-EMR) on passive avoidance behaviour and hippocampal morphology in Wistar rats

**DOI:** 10.3109/03009730903552661

**Published:** 2010-04-07

**Authors:** Sareesh Naduvil Narayanan, Raju Suresh Kumar, Bhagath Kumar Potu, Satheesha Nayak, P. Gopalakrishna Bhat, Maneesh Mailankot

**Affiliations:** ^1^Department of Physiology, Melaka Manipal Medical College, Manipal University, ManipalIndia; ^2^Department of Anatomy, Kasturba Medical College, Manipal University, ManipalIndia; ^3^Department of Anatomy, Melaka Manipal Medical College, Manipal University, ManipalIndia; ^4^Department of Biochemistry, Kasturba Medical College, Manipal University, ManipalIndia; ^5^Department of Biochemistry, Melaka Manipal Medical College, Manipal University, ManipalIndia

**Keywords:** Hippocampus, memory, mobile phone, passive avoidance, RF-EMR (radio-frequency electromagnetic radiation)

## Abstract

**Introduction:**

The interaction of mobile phone radio-frequency electromagnetic radiation (RF-EMR) with the brain is a serious concern of our society.

**Objective:**

We evaluated the effect of RF-EMR from mobile phones on passive avoidance behaviour and hippocampal morphology in rats.

**Materials and methods:**

Healthy male albino Wistar rats were exposed to RF-EMR by giving 50 missed calls (within 1 hour) per day for 4 weeks, keeping a GSM (0.9 GHz/1.8 GHz) mobile phone in vibratory mode (no ring tone) in the cage. After the experimental period, passive avoidance behaviour and hippocampal morphology were studied.

**Results:**

Passive avoidance behaviour was significantly affected in mobile phone RF-EMR-exposed rats demonstrated as shorter entrance latency to the dark compartment when compared to the control rats. Marked morphological changes were also observed in the CA_3_ region of the hippocampus of the mobile phone-exposed rats in comparison to the control rats.

**Conclusion:**

Mobile phone RF-EMR exposure significantly altered the passive avoidance behaviour and hippocampal morphology in rats.

## Introduction

The use of mobile phones is increasing day by day, and it is estimated that approximately 500 million people worldwide are using mobile phones currently. A large proportion of users is made up of children and teenagers. Due to the wide and growing use of mobile communication, there is increasing concern about the interactions of electromagnetic radiation with the human organs and, in particular, with the brain. Experimental studies have shown that the radio-frequency electromagnetic radiation (RF-EMR) emitted from the mobile phones can affect the brain in various ways. These effects have been described *in vitro* and *in vivo* in a number of studies: in particular, effects on cerebral blood flow (1–4), blood-brain barrier permeability (4), oxidant and antioxidant balance (5), neurotransmitter balance (6), nerve cell damage (7), and genomic responses (8) have been reported. There is some concern that short-term memory loss or other cognitive effects may be associated with the use of mobile telephones. In our previous study we had reported that mobile phone exposure in Wistar rats resulted in impaired spatial memory performance in the Morris Water Maze (MWM) test, demonstrated as more time taken to reach the target quadrant and less time spent in the target quadrant (9). In the present study, we tried to evaluate the effect of long-term exposure to RF-EMR emitted from a mobile phone (0.9 GHz/1.8 GHz) on passive avoidance behaviour and hippocampal morphology in male Wistar rats.

## Materials and methods

### Animals

Inbred healthy male albino Wistar rats (8–10 weeks old) were used in this experiment. They were obtained from Manipal University (MU) central animal facility. The rats were housed in plastic cages of size 36 cm × 23 cm × 21 cm (three rats in each cage) inside a temperature- and humidity-controlled environment with free access to food and water *ad libitum*, with a 12 h light and 12 h dark cycle. All the experiments were carried out with prior approval from the institutional animal ethics committee. Care was taken to handle the rats in a humane manner, and all precautions were taken to use the minimum number of animals required to generate significant data.

### Experimental design

Animals were divided into two groups: group I (*n* = 12), normal control; and group II (*n* = 12) were exposed to RF-EMR by giving 50 missed calls (within 1 hour) per day for 4 weeks, keeping a GSM (0.9 GHz/1.8 GHz) mobile phone in vibratory mode (no ring tone) in the cage (9). Each missed call was of the duration of 45 seconds. Animals were free to move in the cage. The phone was kept in a small wood-bottomed cage sized 12 cm × 7 cm × 7 cm. The bamboo wire mesh on top of the wood bottom cage prevented the animals from contact with the phone. Twenty-four hours after the last exposure, six randomly picked animals from both groups were tested for passive avoidance behaviour using passive avoidance apparatus. This test was conducted between 4.00 p.m. and 6.00 p.m. The remaining animals from both groups were sacrificed to study the histological changes in the hippocampus. Statistical analysis was done by using Student's *t* test. *P*-value < 0.05 was considered as significant.

### Passive avoidance apparatus

The apparatus has two compartments, a rectangular larger compartment with a 50 cm × 50 cm grid floor and wooden walls of 35 cm height. It has a roof, which can be opened or closed. One of the walls has a 6 cm × 6 cm opening connecting the larger compartment to a dark smaller compartment. The smaller compartment has a 15 cm × 15 cm electrifiable grid connected to a constant current stimulator, wooden walls of 15 cm height, and a ceiling that can be opened or closed. The connection between the two compartments can be closed with a sliding door made of Plexiglas. The larger compartment was illuminated with a 100-W bulb placed 150 cm above the centre.

### Passive avoidance test

Passive avoidance test was conducted by the method of Bures et al. (10), with modifications. The experiment had three parts: 1) an exploration test, 2) an aversive stimulation and learning test, and 3) a retention test. The exploration test was conducted in three trials. During this, each rat was kept in the centre of the larger compartment facing away from the entrance to the dark compartment. The door between the two compartments was kept open. The rat was allowed to explore the apparatus (both larger and smaller compartments) for 3 minutes. In each trial, the total time taken by the animal to enter the dark compartment was noted using a stop-watch. At the end of the trial, the rat was replaced in the home cage, where it remained during an inter-trial interval of 5 minutes. After the last exploration trial, the rat was again kept in the larger compartment as in the trial sessions. When the animal entered the smaller compartment, the sliding door between the two compartments of the apparatus was closed and three strong foot shocks (50 Hz, 1.5 mA, and 1 s duration) were given at 5-second intervals. The ceiling was then opened and the rat was then returned to its home cage. The retention test was carried out after 24 and 48 hours. The rat was kept in the centre of the larger compartment facing away from the entrance to the smaller compartment for a maximum period of 3 minutes. The sliding door was kept open during this period. The latency time required for the animal to enter the dark compartment was recorded. The latency time was recorded as 3 minutes for those animals that did not enter the dark compartment within 3 minutes. Absence of entry into the dark compartment indicated positive memory retention.

### Hematoxylin and eosin (H&E) staining

All histological procedures were uniform for control and test group animals. The rats were sacrificed by cervical dislocation under ether anaesthesia, and the brain was exposed by cutting the skull along the mid-line. The whole brain was carefully dissected out and fixed in 10% buffered formalin (with pH 7.4) for 24 h. It was then dehydrated in ethanol, defatted in xylene, and embedded in paraffin. Care was taken to ensure that all brains were oriented in the same direction during embedding to minimize differences in the angles at which the brains were sectioned. A single investigator processed all brains to maintain consistency in histological analysis. Sections were cut on a rotary microtome (Leica RM2155, Germany) at 5-micron thickness and stained with hematoxylin and eosin (H&E) according to standard procedure. The hippocampal CA_3_ region was studied under a light microscope. To avoid observer's bias, an independent person coded the slides before subjecting them to morphological evaluations.

## Results

### Passive avoidance test

**Figure 1.**
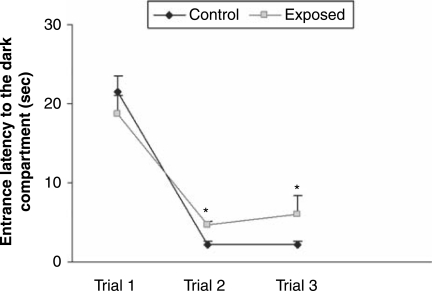
Time taken by the animals to enter the dark compartment of the passive avoidance apparatus during the exploration trials of passive avoidance test. The entrance latency to the dark compartment was decreased in both the groups from first to third trial, but there was a significant difference in the entrance latency time of the groups in the second and third trials. The radio-frequency electromagnetic radiation (RF-EMR)-exposed animals took more time to enter the dark compartment during the exploration trials. *P < 0.05.

In the exploration trials, the entrance latency to the dark compartment was decreased in both the groups from first to third trial, but there was a significant difference in the entrance latency time of the groups in the second and third trials. The RF-EMR-exposed animals took more time to enter the dark compartment during the second and third exploration trials (Figure 1).

**Figure 2.**
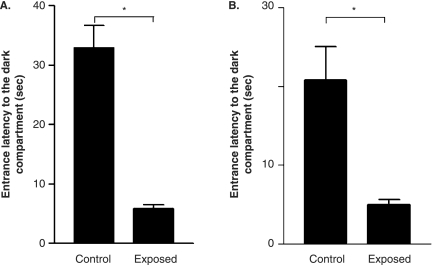
Effect of radio-frequency electromagnetic radiation (RF-EMR) on latency to enter the dark compartment 24 hours (A) and 48 hours (B) after the shock trial. Rats exposed to the mobile phone took significantly less time to enter the dark compartment in the memory retention test. Results are expressed as mean ± SEM. *P < 0.05.

During the memory retention test, the entrance latency to the dark compartment was significantly less for mobile phone-exposed rats when compared with the control group. The latency was approximately four times less in the mobile phone-exposed animals tested 24 hrs after the shock trial (Figure 2A), and the latency was approximately three times less in the mobile phone-exposed rats tested 48 hours after the shock trial (Figure 2B).

### Hippocampal morphology

**Figure 3.**
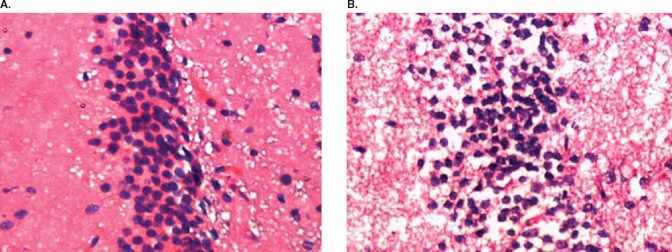
Representative photomicrograph of sections of hippocampal CA_3_ region of the brain from both control and radio-frequency electromagnetic radiation (RF-EMR)-exposed rat stained with hematoxylin and eosin. A: Control animal; row of normal nerve cells in a section of the pyramidal cell band of the hippocampus CA_3_ region is seen. B: Mobile phone RF-EMR-exposed rat; among the normal nerve cells, dark (deeply stained) and shrunken nerve cells are seen.

In comparison to the control animals, marked morphological changes were detected in the CA_3_ region of the hippocampus of the RF-EMR-exposed rats. The hippocampus of RF-EMR-exposed rats showed shrunken, darkly stained neurons (Figure 3B). No such changes were observed in the control rats (Figure 3A).

## Discussion

Passive avoidance tests or conditioned avoidance tests have been used in several studies to assess memory or retention and also retrieval after or during other treatments (11–13). Generally rats avoid intense illumination and prefer dim illumination. When placed in a brightly illuminated space connected with a dark enclosure, they rapidly enter the dark compartment and remain there. After an aversive consequence (foot shock) in the dark compartment, the animals modify their behaviour by inhibiting the innate activities or learned habits (staying in the dark) and remain in the bright compartment (10). So, in this task the animals learn to avoid a noxious event by suppressing a particular behaviour (14).

In the current study, the mobile phone exposure significantly affected the passive avoidance behaviour in rats. In other words, the memory retention and the retrieval were significantly affected in mobile phone RF-EMR-exposed rats. In comparison to the control group, mobile phone-exposed animals showed shorter latency to enter into the dark compartment in the memory retention test (24 h and 48 h after the aversive stimulus). This showed that the animals, after being exposed to aversive stimulation (foot shock) in the passive avoidance task, did not remember this task to some extent on the following day, and this clearly indicates the impairment of the memory. In mobile phone-exposed animals the associative memory which had built up through repetition over many trials and expressed primarily in the performance of tasks was affected. This change in the behaviour of animals (the shorter latency to enter the dark compartment) in the passive avoidance task could be due to the altered functioning of both hippocampal and amygdaloidal neurons due to the damage caused by the RF-EMR emitted from the mobile phone. A number of clinical and experimental studies have shown the role of hippocampal formation and related structures in the medial temporal lobe in learning and memory (15,16). In rats, bilateral lesion of the specific areas of the hippocampus (CA_1_ and CA_3_) produced greater impairments in the performance of passive avoidance task (17). Bilateral hippocampal lesions in chicks caused decreased retention of the avoidance response (18). These studies suggest the involvement of the hippocampal system in associative learning processes and in memory.

In our current study, the hematoxylin and eosin staining of the hippocampal region clearly showed interspersed, deeply stained, shrunken cells, which clearly indicates the degenerative changes in these areas. The exact mechanism responsible for this degeneration has to be investigated; perhaps the mechanism is through reactive oxygen species. Earlier reports have stated that mobile phones caused oxidative damage biochemically by increasing the levels of Malondialdehyde (MDA), carbonyl groups, Xanthine oxidase (XO) activity, and decreasing CAT activity; and that treatment with melatonin significantly prevented oxidative damage in the brain (19). The studies on guinea-pigs have shown increases in MDA, vitamins A, D_3_ (3), and E levels, increased CAT enzyme activity, and decreased Glutathione (GSH) level in the blood of Electromagnetic field (EMF)-exposed guinea-pigs (20). The rats, when exposed to 900 MHz electromagnetic radiation from a mobile phone for 7 days (1 h/day) showed 1) increase in malondialdehyde and nitric oxide levels in brain tissue, 2) decrease in brain superoxide dismutase and glutathione peroxidase activities, and 3) increase in brain xanthine oxidase and adenosine deaminase activities. *Ginkgo biloba* significantly prevented these changes in the brain (21). Exposure of adult Sprague-Dawley rats to regular cell phones resulted in mRNA up-regulation of several injury-associated proteins, such as calcium ATPase, neural cell adhesion molecule, neural growth factor, and vascular endothelial growth factor (22). The possible role of programmed cell death in the brain could also not to be excluded. Short-term exposure to cell phone radio-frequency emissions (1900 MHz) can up-regulate elements of apoptotic pathways in cells derived from the brain, and neurons appear to be more sensitive to this effect than are astrocytes (23). The primary neuronal cultures of rats exposed to a continuous wave (CW) 900 MHz Radiofrequency fields (RF) for 24 h induced apoptosis through a caspase-independent pathway that involves Apoptosis inducing factor (AIF) (24).

Both neurons and glia interact dynamically to enable information processing and behaviour (25,26). The poor performance of rats in the behavioural tests could also be due to the damaging effect of microwaves on glial cells, which in turn alters the neuronal activity in the rat hippocampus and amygdala. Acute exposure to GSM 900 MHz electromagnetic fields (a single GSM exposure = 15 min) induced glial reactivity and biochemical modifications in the rat brain (27). Chronic exposure to GSM 900 MHz microwaves induced persistent astroglia activation in the rat brain, which is the sign of a potential gliosis (28). Reports also suggest that both amygdala and hippocampus act synergistically to form long-term memories of significantly emotional events, and these brain structures are activated following an emotional event and cross-talk with each other in the process of consolidation (29). In order to prove the involvement of various pathways (Reactive Oxygen Species (ROS), apoptosis, or glial reactivation, or a combination of all three) in the alteration of rat behaviour and hippocampal morphology after mobile phone RF-EMR exposure, further studies are warranted.

## Conclusion

The health effects of commonly encountered radio-frequency electromagnetic radiations (RF-EMR) from mobile phone exposures do exist. The evidence from this study points to the quite substantial hazard of RF-EMR from the mobile phone on passive avoidance behaviour and hippocampal morphology in rats.
